# Effects of Cholinergic Neuromodulation on Thalamocortical Rhythms During NREM Sleep: A Model Study

**DOI:** 10.3389/fncom.2019.00100

**Published:** 2020-01-23

**Authors:** Qiang Li, Jiang-Ling Song, Si-Hui Li, M. Brandon Westover, Rui Zhang

**Affiliations:** ^1^Medical Big Data Research Center, Northwest University, Xi'an, China; ^2^Department of Neurology, Massachusetts General Hospital, Boston, MA, United States

**Keywords:** thalamocortical neural mass model, cholinergic projection, acetylcholine (ACH), thalamocortical rhythm, NREM sleep

## Abstract

It has been suggested that cholinergic neurons shape the oscillatory activity of the thalamocortical (TC) network in behavioral and electrophysiological experiments. However, theoretical modeling demonstrating how cholinergic neuromodulation of thalamocortical rhythms during non-rapid eye movement (NREM) sleep might occur has been lacking. In this paper, we first develop a novel computational model (TC-ACH) by incorporating a cholinergic neuron population (CH) into the classical thalamo-cortical circuitry, where connections between populations are modeled in accordance with existing knowledge. The neurotransmitter acetylcholine (ACH) released by neurons in CH, which is able to change the discharge activity of thalamocortical neurons, is the primary focus of our work. Simulation results with our TC-ACH model reveal that the cholinergic projection activity is a key factor in modulating oscillation patterns in three ways: (1) transitions between different patterns of thalamocortical oscillations are dramatically modulated through diverse projection pathways; (2) the model expresses a stable spindle oscillation state with certain parameter settings for the cholinergic projection from CH to thalamus, and more spindles appear when the strength of cholinergic input from CH to thalamocortical neurons increases; (3) the duration of oscillation patterns during NREM sleep including K-complexes, spindles, and slow oscillations is longer when cholinergic input from CH to thalamocortical neurons becomes stronger. Our modeling results provide insights into the mechanisms by which the sleep state is controlled, and provide a theoretical basis for future experimental and clinical studies.

## 1. Introduction

Sleep plays a pivotal role in mental and physical health. During sleep, the brain alternates between two stages, rapid-eye movement (REM) and NREM. This alternation is reflected in the electrical rhythms generated by the thalamocortical system, evident in the electroencephalogram (EEG). Specifically, the thalamocortical rhythms in NREM sleep include K-complexes and slow oscillations (dominated by low-frequency [0.5, 2]Hz, high amplitude oscillations), as well as spindles (characterized by a waxing and waning waveform in the range [11, 16]Hz). In contrast, REM sleep exhibits low amplitude activity of higher frequency, which is similar with rhythms in wakefulness, including the alpha oscillation (Rasch and Born, 2013). Studies reveal that spindles and slow oscillations are very helpful in protecting and stabilizing sleep (Roth, 2009; Dang-Vu et al., 2010; Kim et al., 2012).

A number of results in behavioral and electrophysiological experiments suggest that cholinergic neurons, which are distributed in the basal forebrain and brainstem, help shape the oscillatory activity of the thalamocortical network (Bellingham and Funk, 2000; Kobayashi et al., 2003; Boutrel and Koob, 2004; Brown et al., 2012). Meanwhile, it has also been shown that thalamocortical processing is subject to the action of modulatory of acetylcholine (ACH) released by cholinergic neurons (McCormick, 1992). However, a theoretical framework for understanding the mechanisms of cholinergic neuromodulation on thalamocortical rhythms during NREM sleep is still lacking.

Neural mass models (NMMs), which describe the dynamics of neural populations (Wilson and Cowan, 1973; Lopes da Silva et al., 1974), have shown success in clarifying the mechanisms involved in the generation and regulation of oscillation patterns of the “wakefulness-sleep cycle.” For example, Robinson et al. (2002) developed a continuum model of large-scale brain electrical activity to understand the transition from the resting background state to the spike-wave state. Suffczynski et al. (2004) proposed a thalamocortical NMM to explain the relation between mechanisms that generate sleep spindles and those for that generate spike-wave activity. More recently, Weigenand et al. (2014) extended a cortical NMM to explore the mechanisms for generating K-complexes and slow oscillations. Cona et al. (2014) presented a new NMM to describe the sleeping thalamocortical system, where thalamic neurons exhibit two firing modes: bursting and tonic.

Although prior (NMM-based) studies have made progress in explaining sleep rhythms, the role of ACH modulation of thalamocortical activities, especially during sleep has not been thoroughly explored. In this work we model the details of cholinergic neuromodulation in the cortex and thalamus, and study the effects of cholinergic projections on thalamocortical rhythms during NREM sleep.

In this study, a new computational model (TC-ACH) is first proposed by integrating a cholinergic neuron population (CH) into classical thalamo-cortical circuitry, with connections between populations in TC-ACH constructed on the basis of the existing mechanisms. We concentrate on the neurotransmitter ACH released by cholinergic neurons, which impacts firing activity of thalamocortical neurons. The proposed model TC-ACH is used to investigate effects of ACH modulation on thalamocortical rhythms during NREM sleep in two ways: (1) measurement of effects of cholinergic modulation in the thalamus; (2) measurement of effects of cholinergic modulation in the thalamocortical system.

The rest of the paper is organized as follows. In section 2, the model framework and mathematical expression of TC-ACH are introduced. Section 3 presents the simulation results. A summary and discussion of our work are given in the last section.

## 2. Methods

### 2.1. Model Framework

This subsection introduces the topological structure and computational operators of TC-ACH systematically.

#### 2.1.1. Topological Structure

**Figure 1:**
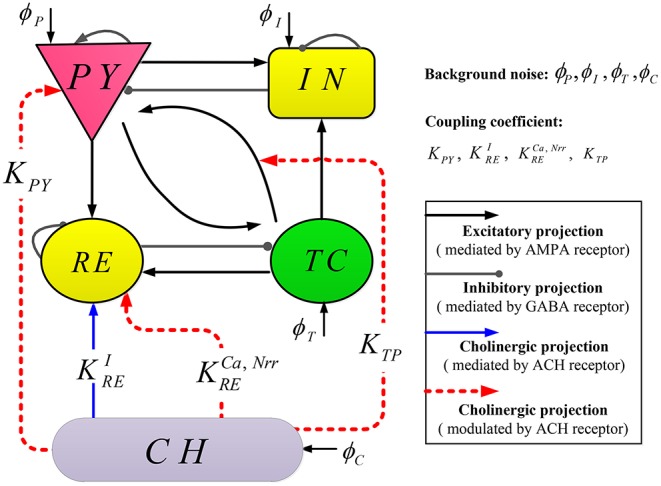
Schematic representation of model TC-ACH.

Our computational model TC-ACH is composed of classical thalamo-cortical circuitry as well as a cholinergic neuron population, whose topological structure is illustrated in Figure 1.

The thalamo-cortical circuitry consists of two mutually interconnected modules: a cortical module and a thalamic module. Each module comprises two neural populations, where pyramidal cells (PY) and inhibitory interneurons (IN) are included in the cortical module; the thalamic module also includes thalamocortical cells (TC) and thalamic reticular cells (RE). Regarding connections between modules, excitatory neurons (PY or TC) in each module connect to both populations in the other module. Within each module, two populations project to each other, and there exist self-connections within PY, IN, and RE (Costa et al., 2016). Note that in the thalamo-cortical circuitry, the excitatory and inhibitory projections are mediated by AMPA and GABA receptors, respectively.

The cholinergic neuron population (CH) is located in the basal forebrain and brainstem. The neurons in CH release neurotransmitter (ACH) into the synaptic cleft, which changes the activity of thalamocortical neurons. In our proposed model, there exist three modulated cholinergic projections from CH to PY, RE, as well as the connection between TC and PY (represented by red dashed lines in Figure 1), and one mediated cholinergic projection from CH to RE (represented by blue the solid line in Figure 1). The coupling coefficients of these projections are denoted by *K*_*PY*_, $K_{RE}^{Nrr}$, $K_{RE}^{Ca}$, *K*_*TP*_, and $K_{RE}^{I}$, respectively. Here, all projections involve by ACH.

#### 2.1.2. Computational Operators

The dynamical evolution in each population is implemented in two computational blocks.

The first computational block transforms the average membrane potential *V*(*t*) into the average firing rate *Q*(*t*), formulated by a sigmoid function with the form (Jansen et al., 1993)

(1) $$\begin{array}{l} Q\left(t\right)=\frac{Q^{max}}{1+e^{-\left(V\left(t\right)-\theta \right)\;/\sigma }}, \end{array}$$

where *Q*^*max*^, θ, σ represent the maximal firing rate, the firing threshold and the neural gain, respectively.

In the second computational block, the firing rate *Q*(*t*) is first transformed into the fraction of open channels *r*_ξ_(*t*) by a convolution with an alpha function *h*_ξ_(*t*), that is,

(2) $$\begin{array}{l} r_{\xi }\left(t\right)=h_{\xi }\left(t\right)⊗\left(N\cdot Q\left(t\right)\right), \end{array}$$

(3) $$\begin{array}{l} h_{\xi }\left(t\right)=\gamma_{\xi }^{2}\cdot te^{-\gamma_{\xi }t},t\geq 0. \end{array}$$

Then the membrane potential *V*(*t*) is solved by

(4) $$\begin{array}{lll} \tau \dot{V}\left(t\right) & = & -I_{L}\left(t\right)-I_{AMPA}\left(t\right)-I_{GABA}\left(t\right)\\ \; & = & -{\bar{g}}_{L}\cdot \left(V\left(t\right)-E_{L}\right)\\ \; & \; & -\underset{\xi }{\sum }{\bar{g}}_{\xi }r_{\xi }\cdot \left(V\left(t\right)-E_{\xi }\right), \end{array}$$

which is similar to the classical conductance-based form of Hodgkin and Huxley (1952) with one leak and two synaptic currents (say, ξ ∈ {*AMPA, GABA*}) (Weigenand et al., 2014). Moreover, for the convenience of calculating the convolution ⊗, *r*_ξ_(*t*) can be equivalently obtained by solving the following second-order differential equation

(5) $$\begin{array}{l} {\ddot{r}}_{\xi }\left(t\right)=\gamma_{\xi }^{2}\cdot \left(N\cdot Q\left(t\right)-r_{\xi }\left(t\right)\right)-2\gamma_{\xi }{\dot{r}}_{\xi }\left(t\right). \end{array}$$

**Figure 2:**
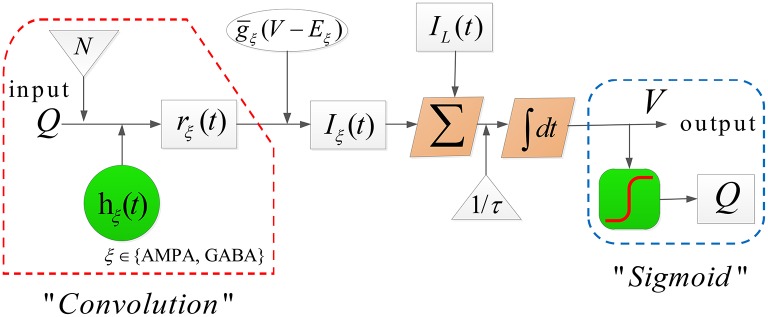
The dynamical evolution in each population in model TC-ACH.

In (2)-(5), *E*_ξ_ and *E*_*L*_ denote the reversal potential of the synaptic current and leak current, respectively, ${\bar{g}}_{\xi }$ represents the synaptic input rate that scales *r*_ξ_ and *E*_ξ_, *g*_*L*_ is the maximal conductivity of the leak current conductance, τ is the membrane time constant, *N* stands for the connectivity constant, and γ_ξ_ is the rate constant of synaptic response. The detailed dynamical evolution in each population is illustrated in Figure 2.

### 2.2. Mathematical Expression

This subsection formulates the proposed model TC-ACH mathematically in the logic of five populations included in the model.

#### 2.2.1. Cholinergic Neuron Population (CH)

We first consider the cholinergic neuronal population, which is an essential part of TC-ACH in studying the effects of cholinergic modulation on thalamocortical rhythms during NREM sleep. Due to the special role of ACH, here, we apply the concentration of ACH ([*ACH*]) rather than the firing rate applied in Equation (1) to complete the transformation from the membrane potential, with Destexhe et al. (1994)

(6) $$\begin{array}{l} \left[ACH\right]\left(t\right)=\frac{{\left[ACH\right]}^{max}}{1+e^{-\left(V_{c}\left(t\right)-\theta_{c}\right)\;/\sigma_{c}}}, \end{array}$$

where [*ACH*]^*max*^ is the maximum concentration of ACH.

Moreover, CH receives an external input current *I*_*ext*_. Therefore, the membrane potential *V*_*c*_(*t*) in CH obeys

(7) $$\begin{array}{l} \tau_{c}{\dot{V}}_{c}\left(t\right)=-I_{L}^{c}\left(t\right)-I_{ext}\left(t\right), \end{array}$$

here, *I*_*ext*_ has the form (Rudolph et al., 2004)

(8) $$\begin{array}{l} I_{ext}=g_{ext}\left(t\right)\left(V_{c}\left(t\right)-E_{c}\right), \end{array}$$

(9) $$\begin{array}{l} \dot{g_{ext}}\left(t\right)=\frac{-\left(g_{ext}\left(t\right)-g_{0}\left(t\right)\right)}{\tau_{ext}}+\sqrt{\frac{2\sigma_{ext}^{2}}{\tau_{ext}}}\phi_{C}\left(t\right), \end{array}$$

where *g*_0_ is the average conductance, τ_*ext*_ is the time constant, σ_*ext*_ is the noise standard deviation (SD) value, and ϕ_*C*_(*t*) denotes the independent Gaussian white noise process of unit SD and zero mean.

#### 2.2.2. Populations PY and IN in the Cortical Module

According to Clearwater et al. (2008), we know that ACH acts on PY both pre-synaptically and post-synaptically over time scales from milliseconds to minutes.

In the post-synaptic case, ACH acts on PY through muscarinic receptors, which can cause a certain suppression of voltage-gated potassium channels (McCormick, 1992). Hence, the M-type potassium current $I_{M}^{p}$ generated by the inactivation of these channels will change the intrinsic excitability of PY. Motivated by this observation, the membrane potential *V*_*p*_(*t*) in PY is then formulated by

(10) $$\begin{array}{l} \tau_{p}{\dot{V}}_{p}\left(t\right)=-I_{L}^{p}\left(t\right)-I_{AMPA}^{p}\left(t\right)-I_{GABA}^{p}\left(t\right)-I_{M}^{p}\left(t\right), \end{array}$$

where $I_{L}^{p},I_{AMPA}^{p},I_{GABA}^{p}$ are calculated by Equations (2)–(4). The newly added current $I_{M}^{p}$ is described as follows (Rich et al., 2018)

(11) $$\begin{array}{l} \;I_{M}^{p}\left(t\right)=g_{M}\left(t\right)\cdot \left(V_{p}\left(t\right)-E_{M}\right)z\left(t\right), \end{array}$$

where *z*(*t*) represents the unitless gating variable of the ionic current conductance satisfying

(12) $$\begin{array}{l} \dot{z}\left(t\right)=\frac{\bar{z}\left(t\right)-z\left(t\right)}{\tau_{z}}. \end{array}$$

Here, $\bar{z}\left(t\right)=\frac{1}{1+e^{\left(-V_{p}\left(t\right)-39\right)/5}}$ stands for the voltage-sensitive steady-state activation function and τ_*z*_ is the time constant. Note that *g*_*M*_ in Equation (11) is not a constant anymore, but varies in term of [*ACH*], that is,

(13) $$\begin{array}{l} \;g_{M}\left(t\right)=g_{M}^{*}+K_{PY}\cdot \left[ACH\right]\left(t\right), \end{array}$$

where $g_{M}^{*}$ is a constant with the nominal value listed in Table 1.

**Table 1:** Description and nominal values of parameters in model TC-ACH.

**Symbol**	**Description**	**Value**	**Unit**
*C*_*m*_	Membrane capacitance in the HH model	1	μ*F*/*cm*^2^
$Q_{p}^{max}$		30·10^−3^	*ms*
$Q_{i}^{max}$	Maximal firing rate	60·10^−3^	
$Q_{t}^{max},Q_{r}^{max}$		400·10^−3^	
[*ACH*]^*max*^	Maximum [ACH] in the synaptic cleft	1	*mM*
θ	Firing threshold	−58.5	*mV*
σ_*p*_	Inverse neural gain	4.7	*mV*
σ_*i*_, σ_*t*_, σ_*r*_		6	
θ_*c*_	[ACH] threshold	−40	*mV*
σ_*c*_	The steepness of the sigmoid	4	*mV*
σ_*ext*_	The noise standard deviation	12	*nS*
γ_*e*_		70·10^−3^	*ms*^−1^
γ_*g*_	Synaptic rate constant	58.6·10^−3^	
γ_*r*_, γ_*c*_		100·10^−3^	
*N*_*pp*_, *N*_*ip*_, *N*_*pi*_, *N*_*ii*_		120, 72, 90, 90	/
$N_{tp}^{*},N_{tp}^{\infty }$		2.5, 5.5	
*N*_*rp*_, *N*_*rt*_, *N*_*tr*_	Connectivity constant	2.6, 3, 5	
${\bar{N}}_{rr}$		25	
*N*_*pt*_, *N*_*it*_		2.5	
τ_*p*_, τ_*i*_		30	*ms*
τ_*t*_, τ_*r*_, τ_*c*_	Membrane time constant	20	
τ_*z*_, τ_*ext*_		75, 2.73	
${\bar{g}}_{x\in \left\{AMPA,GABA\right\}}$	Input rate of synaptic channel	1	*ms*
$g_{M}^{*},g_{T}^{t},g_{h}$		1.6, 3, 0.62	*mS*/*cm*^2^
*g*_*LK*_	Conductivity of ion channel	0.18−0.55	
${\bar{g}}_{T}^{r}$	Maximum calcium conductivity	3	*mS*/*cm*^2^
*g*_*inc*_	Conductivity scaling of h-current	2	/
*g*_*ACH*−*n*_	Conductance mediated by nACHR	0.15	*mS*/*cm*^2^
*g*_0_	Average conductance	12.1	*nS*
$E_{L}^{p},E_{L}^{i}$		−64	*mV*
$E_{L}^{t},E_{L}^{r},E_{L}^{c},E_{c}$		−70	
*E*_*LK*_, *E*_*Ca*_, *E*_*h*_	Nernst reversal potential	−100, 120, −40	
*E*_*AMPA*_, *E*_*ACH*−*n*_		0	
*E*_*GABA*_		−70	
μ	Concentration constant	1	*mM*
*a, b, c*	Positive constant	0.05, 0.58, 0.42	/
ω_*max*_	Maximum conductance	0.96	*mS*/*cm*^2^
*P*_*d*_	Apparent dissociation constant	0.028	*mM*
*h*	Hill coefficient of ACH binding to the receptors	1.8	/
*v*	Axonal rate constant	120·10^−3^	*ms*^−1^
ϕ_0_	Mean background noise	0	*ms*^−1^
$\phi_{P}^{sd},\phi_{I}^{sd}$	Standard deviation of cortical background noise	120·10^−3^	
$\phi_{T}^{sd}$	Standard deviation of thalamic background noise	10·10^−3^	
*K*_*i* ∈ {*PY, TP*}_	Coupling coefficient of projection from CH	1.5−5.5	/
$K_{RE}^{j\in \left\{Ca,Nrr,I\right\}}$		1−9	

In the pre-synaptic case, ACH acts on PY to modulate the properties of thalamocortical synapses via nicotinic receptor, which can result in changing the thalamocortical synaptic connection strength from TC to PY (denoted by *N*_*tp*_) (Gil et al., 1997). Furthermore, as summarized by Kimura (2000), the thalamocortical connection strength will increase when the value of [*ACH*] becomes larger. Hence, we apply the way in Clearwater et al. (2008) to reformulate the connection strength *N* in Equation (5) as

(14) $$\begin{array}{l} N_{tp}\left(t\right)=\left(N_{tp}^{*}-N_{tp}^{\infty }\right)e^{\frac{-K_{TP}\cdot \left[ACH\right]\left(t\right)}{\mu }}+N_{tp}^{\infty }. \end{array}$$

Obviously, there have

$$N_{tp}\left(t\right)=\{\begin{array}{cc} N_{tp}^{\infty }, & \text{as }\left[ACH\right]\left(t\right)\to \infty \text{;}\\ N_{tp}^{*}, & \text{as }\left[ACH\right]\left(t\right)\to 0\text{.} \end{array}$$

where $N_{tp}^{\infty }$ and N^*^ represent the connectivity values under two extreme situations, and μ is a concentration constant.

Because in the cortical module IN contributes less than PY to the effects of cholinergic modulation on thalamocortical rhythms (Picciotto et al., 2012), in this paper, the dynamic evolution in IN is assumed to be not affected by [*ACH*]. That is to say, the mathematical formulation of IN obeys Equations (1)–(5).

#### 2.2.3. Populations RE and TC in Thalamic Module

We see in Figure 1 that population RE appears to be a central hub in connecting the cortex and population CH. The electrical activity occurring in RE is important for regulating information transmission in the thalamocortical system and the shape of thalamocortical rhythms. Therefore, we first model the discharge activity of RE in this section.

On one hand, recent research indicates that thalamic reticular neurons can be directly excited by activating α7-containing nicotinic ACH receptor (Ni et al., 2016). Consequently, we introduce a new current *I*_*ACH*−*n*_ as an input of RE from CH (corresponding the blue solid line in Figure 1).

On the other hand, it has been shown that the potassium leak current and T-type calcium current are essential for the generation of spindle oscillation (Langdon et al., 2012). Based on this fact, two more currents *I*_*LK*_ and *I*_*T*_ are considered in the formulation of the membrane potential *V*_*r*_(*t*) in RE simultaneously.

Hence, the final equation is formulated by

(15) $$\begin{array}{ll} \tau_{r}{\dot{V}}_{r}(t) & =-I_{L}^{r}\left(t\right)-I_{AMPA}^{r}\left(t\right)-I_{GABA}^{r}\left(t\right)-I_{ACH-n}\left(t\right)\\ \; & -C_{m}^{-1}\tau_{r}\cdot \left(I_{LK}^{r}\left(t\right)-I_{T}^{r}\left(t\right)\right). \end{array}$$

In Equation (15), $I_{ACH-n}^{r}$, $I_{LK}^{r}$ and $I_{T}^{r}$ are defined, respectively, as

(16) $$\begin{array}{l} \;I_{ACH-n}\left(t\right)=g_{ACH-n}\cdot \left(V_{r}\left(t\right)-E_{ACH-n}\right)\omega \left(t\right) \end{array}$$

where ω(*t*) is solved by

(17) $$\begin{array}{l} \dot{\omega }(t)=\frac{\omega_{max}}{1+{\left(\frac{\;P_{d}}{\;K_{RE}^{I}\cdot [ACH](t)}\right)}^{h}}, \end{array}$$

(18) $$\begin{array}{l} I_{LK}^{r}=g_{LK}^{r}\cdot \left(V_{r}\left(t\right)-E_{LK}^{r}\right), \end{array}$$

(19) $$\begin{array}{l} I_{T}^{r}\left(t\right)=g_{T}^{r}\left(t\right)\cdot \left(V_{r}\left(t\right)-E_{Ca}^{r}\right){\left(m\left(t\right)\right)}^{2}h\left(t\right). \end{array}$$

Here, ω denotes the proportion of open ion-channels caused by binding of ACH. *g*_*ACH*−*n*_ and *E*_*ACH*−*n*_ stand for the conductance and reversal potential, respectively. *P*_*d*_ and *h* represent the apparent dissociation constant and Hill coefficient of ACH binding to the receptors, and ω_*max*_ is the maximum conductance. *m*(*t*) and *h*(*t*) are activation and inactivation functions of T-type current. Details about Equations (16)–(19) can be seen in Baran et al. (2010), Sethuramanujam et al. (2016), and Destexhe et al. (1993). In addition, the calcium conductance $g_{T}^{r}$ in Equation (19) is recognized to be important for generating bursting oscillations in RE, whose value increases with increasing [*ACH*] (Fisher and Johnston, 1990). Specifically, it is expressed by

(20) $$\begin{array}{l} \;g_{T}^{r}\left(t\right)={\bar{g}}_{T}^{r}\frac{\;K_{RE}^{Ca}\cdot \left[ACH\right]\left(t\right)}{\;K_{RE}^{Ca}\cdot \left[ACH\right]\left(t\right)+a} \end{array}$$

where ${\bar{g}}_{T}^{r}$ and *a* are positive constants (Omori and Horiguchi, 2004).

In addition, it has been found that the GABAergic projection within RE is decreased by increasing [*ACH*] (Fisher and Johnston, 1990). This mechanism is then absorbed into defining the self-feedback connectivity of RE in our work and formulated by

(21) $$\begin{array}{l} \;N_{rr}\left(t\right)=\left(-b\cdot logK_{RE}^{Nrr}\cdot \left[ACH\right]\left(t\right)+c\right)\cdot {\bar{N}}_{rr}, \end{array}$$

where ${\bar{N}}_{rr}$, *b* and *c* are positive constants (Omori and Horiguchi, 2004).

(22) $$\begin{array}{l} \tau_{p}{\dot{V}}_{p}=-I_{L}^{p}-I_{AMPA}^{p}\left(r_{ep}\right)-I_{GABA}^{p}\left(r_{gp}\right)-C_{m}^{-1}\tau_{p}I_{M} \end{array}$$

(23) $$\begin{array}{l} \tau_{i}{\dot{V}}_{i}=-I_{L}^{i}-I_{AMPA}^{i}\left(r_{ei}\right)-I_{GABA}^{i}\left(r_{gi}\right) \end{array}$$

(24) $$\begin{array}{lll} \tau_{t}{\dot{V}}_{t} & = & -I_{L}^{t}-I_{AMPA}^{t}\left(r_{et}\right)-I_{GABA}^{t}\left(r_{gt}\right)-C_{m}^{-1}\tau_{t}\\ \; & \; & \left(I_{LK}^{t}-I_{T}^{t}-I_{h}\right) \end{array}$$

(25) $$\begin{array}{lll} \tau_{r}{\dot{V}}_{r} & = & -I_{L}^{r}-I_{AMPA}^{r}\left(r_{er}\right)-I_{GABA}^{r}\left(r_{gr}\right)-I_{ACH-n}\\ \; & \; & -C_{m}^{-1}\tau_{r}\left(I_{LK}^{r}-I_{T}^{r}\right) \end{array}$$

(26) $$\begin{array}{l} \tau_{c}{\dot{V}}_{c}=-I_{L}^{c}-I_{ext} \end{array}$$

(27) $$\begin{array}{l} {\ddot{r}}_{ep}=\gamma_{e}^{2}\left(N_{pp}Q_{p}+N_{tp}\eta_{t}+\phi_{P}-r_{ep}\right)-2\gamma_{e}{\dot{r}}_{ep} \end{array}$$

(28) $$\begin{array}{l} {\ddot{r}}_{ei}=\gamma_{e}^{2}\left(N_{pi}Q_{p}+N_{ti}\eta_{t}+\phi_{I}-r_{ei}\right)-2\gamma_{e}{\dot{r}}_{ei} \end{array}$$

(29) $$\begin{array}{l} {\ddot{r}}_{et}=\gamma_{e}^{2}\left(N_{pt}\eta_{p}+\phi_{T}-r_{et}\right)-2\gamma_{e}{\dot{r}}_{et} \end{array}$$

(30) $$\begin{array}{l} {\ddot{r}}_{er}=\gamma_{e}^{2}\left(N_{tr}Q_{t}+N_{pr}\eta_{p}-r_{er}\right)-2\gamma_{e}{\dot{r}}_{er} \end{array}$$

(31) $$\begin{array}{l} {\ddot{r}}_{gp}=\gamma_{g}^{2}\left(N_{ip}Q_{i}-r_{gp}\right)-2\gamma_{g}{\dot{r}}_{gp} \end{array}$$

(32) $$\begin{array}{l} {\ddot{r}}_{gi}=\gamma_{g}^{2}\left(N_{ii}Q_{i}-r_{gi}\right)-2\gamma_{g}{\dot{r}}_{gi} \end{array}$$

(33) $$\begin{array}{l} {\ddot{r}}_{gt}=\gamma_{r}^{2}\left(N_{rt}Q_{r}-r_{gt}\right)-2\gamma_{r}{\dot{r}}_{gt} \end{array}$$

(34) $$\begin{array}{l} {\ddot{r}}_{gr}=\gamma_{r}^{2}\left(N_{rr}Q_{r}-r_{gr}\right)-2\gamma_{r}{\dot{r}}_{gr} \end{array}$$

(35) $$\begin{array}{l} \ddot{\eta_{p}}=v^{2}\left(Q_{p}-\eta_{p}\right)-2v{\dot{\eta }}_{p} \end{array}$$

(36) $$\begin{array}{l} \ddot{\eta_{t}}=v^{2}\left(Q_{t}-\eta_{t}\right)-2v{\dot{\eta }}_{t} \end{array}$$

Next, we turn to modeling the discharge activity of TC. Different from RE, there exists one more *h*-type current in TC, which is responsible for the waxing and waning structure of spindle rhythms in thalamus. Hence, the membrane potential *V*_*t*_(*t*) in TC is formulated by

(37) $$\begin{array}{ll} \tau_{t}V_{t}^{˙}(t) & =-I_{L}^{t}\left(t\right)-I_{AMPA}^{t}\left(t\right)-I_{GABA}^{t}\left(t\right)-C_{m}^{-1}\cdot \tau_{t}\cdot (I_{LK}^{t}\left(t\right)\\ \; & -I_{T}^{t}\left(t\right)-I_{h}\left(t\right)), \end{array}$$

where $I_{LK}^{t},I_{T}^{t}$ are calculated by Equations (18)–(19), and *I*_*h*_ is described as Destexhe et al. (1996)

(38) $$\begin{array}{l} I_{h}\left(t\right)=g_{h}\cdot \left(V_{t}\left(t\right)-E_{h}\right)\left(m_{h1}\left(t\right)+g_{inc}\cdot m_{h2}\left(t\right)\right). \end{array}$$

Here, *g*_*h*_ and *E*_*h*_ represent the conductance and reversal potential, *g*_*inc*_ is the conductivity scaling. The details of functions *m*_*h*1_(*t*) and *m*_*h*2_(*t*) can be found in Destexhe et al. (1993).

#### 2.2.4. Full Mathematical Expression of TC-ACH

There is, to emphasize, one key point in our model. On account of the long range afferent, there exist conduction delays between thalamus and cortex module. According to Costa et al. (2016), this delay is approximated as a convolution with the alpha function *h*(*t*). In this case, Equation (5) is then reformulated by

(39) $$\begin{array}{l} {\ddot{r}}_{\xi }\left(t\right)=\gamma_{\xi }^{2}\left(N\cdot \left(h\left(t\right)⊗Q\left(t\right)\right)-r_{\xi }\left(t\right)\right)-2\gamma_{\xi }{\dot{r}}_{\xi }\left(t\right) \end{array}$$

during four transmissions “PY → RE,” “PY → TC,” “TC → PY,” and “TC → IN.”

The full mathematical expression of TC-ACH is then formulated by Equations (22)–(36) (see page 5). Note that the model output is *V*_*p*_, which can be viewed as the simulated EEG signals.

**Remark 1**. The description and nominal values of all parameters included in the model TC-ACH are listed in Table 1, whose values are the nominal ones reported in Costa et al. (2016), Omori and Horiguchi (2004), Clearwater et al. (2008), Rich et al. (2018), Baran et al. (2010), Sethuramanujam et al. (2016), Bhattacharya et al. (2012), and Rudolph et al. (2004).

## 3. Results

In this section, we apply the proposed model TC-ACH to verify the effects of cholinergic modulation on thalamocortical rhythms during NREM in two ways: (1) measurement of effects of cholinergic modulation in the thalamus; (2) measurement of effects of cholinergic modulation in the thalamocortical system.

All of the numerical simulations are performed in MATLAB R2017b (MathWorks,USA), using a stochastic Runge-Kutta method of 4th order (Rößler, 2010) with a step size of 0.1ms.

### 3.1. The Oscillatory Phenomena Due to Cholinergic Modulation in Thalamus

The thalamus is believed to act as a “pacemaker” for thalamocortical rhythms, and is able to independently generate multiple brain rhythms during sleep (Hughes and Crunelli, 2005; Li et al., 2017). Hence, oscillatory phenomena due to cholinergic modulation in thalamus are first explored, where only the thalamic module and projections from CH to RE are considered. Here, the model output is *V*_*t*_.

We first show different thalamic oscillations and transitions between them caused by cholinergic projections. To this end, we apply one-dimensional bifurcation analysis for several key parameters, represented by *g*_*LK*_, $K_{RE}^{I}$, $K_{RE}^{Ca}$, and $K_{RE}^{Nrr}$. The bifurcation diagram is obtained by plotting the stable local minimum, as well as top three maximum values of *V*_*t*_ over changes in each of four parameters. All simulations are executed for 40 s and those minimum and maximum values are obtained from the latter stable 30 s of the time series.

**Figure 3:**
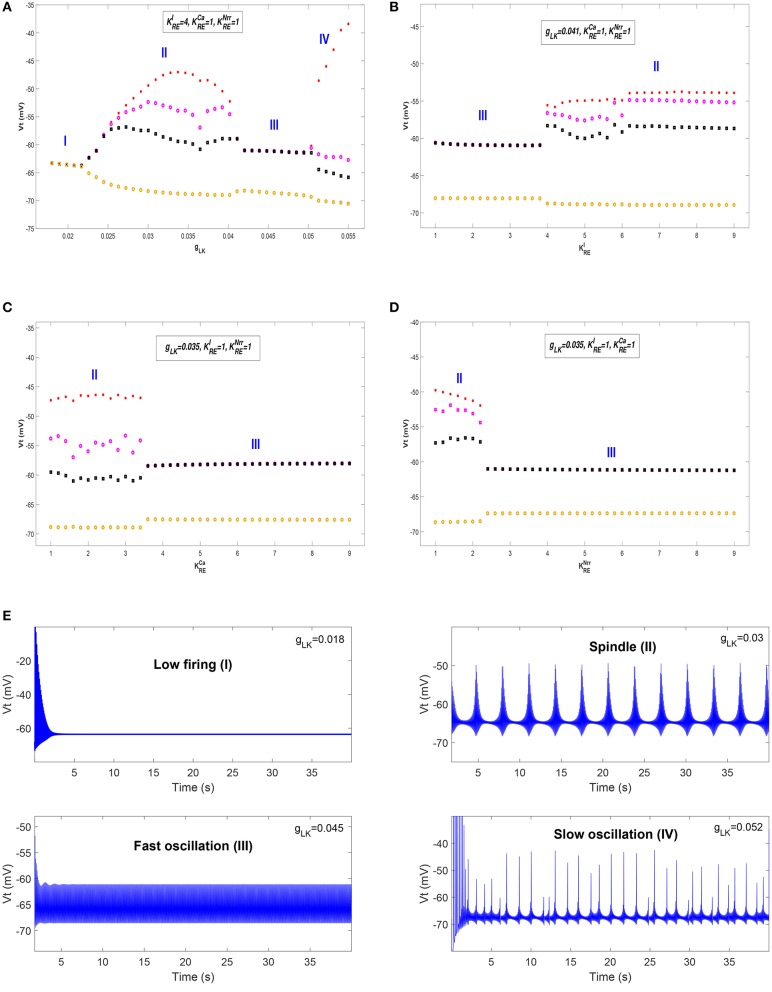
The bifurcation diagrams of *V*_*t*_ over changes in *g*_*LK*_, $K_{RE}^{I}$, $K_{RE}^{Ca}$, and $K_{RE}^{Nrr}$. **(A)** Bifurcation diagram (varying *g*_*LK*_). **(B)** Bifurcation diagram (varying $K_{RE}^{I}$). **(C)** Bifurcation diagram (varying $K_{RE}^{Ca}$). **(D)** Bifurcation diagram (varying $K_{RE}^{Nrr}$). **(E)** Four thalamic oscillations with respect to different values of *g*_*LK*_.

Figures 3A–D shows bifurcation diagrams of *V*_*t*_ over changes in *g*_*LK*_, $K_{RE}^{I}$, $K_{RE}^{Ca}$, and $K_{RE}^{Nrr}$, respectively. Seen from Figure 3A, it reveals that there are four different dynamical states including low firing (I), spindle (II), fast oscillation (III) and slow oscillation (IV) as *g*_*LK*_ varies in [0.018, 0.055]. Specifically, when the value of *g*_*LK*_ is extremely small, the model exhibits a low firing state and no oscillation behavior can be observed. As *g*_*LK*_ becomes a little larger, the model mainly experiences a spindle oscillation pattern for a period, in which multiple pairs of maximum and minimum values are found within each periodic complex. With further growth of *g*_*LK*_, the model exhibits a fast oscillation pattern, in which only one pair of maximum and minimum values emerges within each periodic complex. When strong hyperpolarization through increasing *g*_*LK*_ attains, the model moves from a high frequency oscillation pattern into a slow oscillation pattern, in which multiple pairs of maximum and minimum values can also be observed within each periodic complex, but the distances between the top maximum and other maximum values become larger. Figure 3E illustrates the obtained thalamic oscillations, which correspond to four dynamical states (*g*_*LK*_ = 0.018, 0.03, 0.045, 0.052), respectively.

A similar explanation can be given for the cases of $K_{RE}^{I}$, $K_{RE}^{Ca}$, and $K_{RE}^{Nrr}$, while only two dynamical states (i.e., II and III) are obtained. Moreover, the state transition of $K_{RE}^{I}$ is different from that of $K_{RE}^{Ca}$ and $K_{RE}^{Nrr}$. It is apparent that the model has a pattern transition from state III to an increasingly stable state II as $K_{RE}^{I}$ increases (see Figure 3B). By contrast, a transition from state II to state III with the increase of $K_{RE}^{Ca}$ (or $K_{RE}^{Nrr}$) can be observed in Figure 3C (or Figure 3D). That is to say, the model appears to demonstrate a stabler spindle oscillation state with the increasing value of $K_{RE}^{I}$, while the growth of $K_{RE}^{Ca}$ (or $K_{RE}^{Nrr}$) leads to reduced spindle rhythms. Prior work has established that spindle rhythms are helpful to protect sleep (Dang-Vu et al., 2010; Kim et al., 2012). Therefore, the obtained results implicate that cholinergic modulation in RE may help promote sleep (or arousal) states.

**Figure 4:**
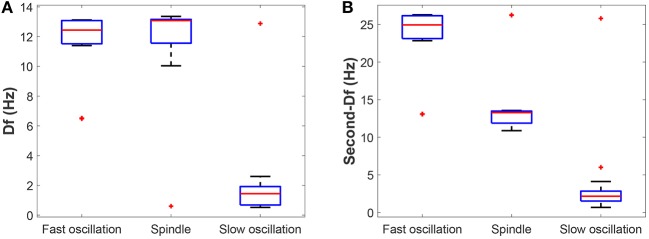
The box-plots of **(A)** Df and **(B)** Sec-Df with respect to three oscillations (fast, slow, and spindle).

**Figure 5:**
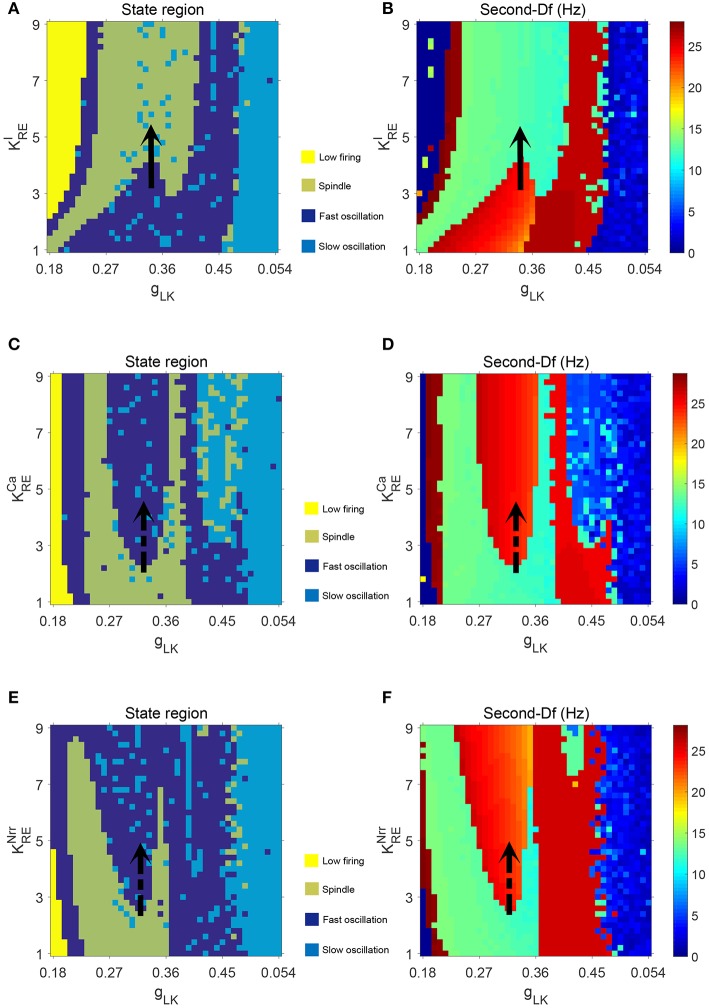
The dynamical state regions **(A,C,E)** and Sec-Df regions **(B,D,F)** in three panels $\left(g_{LK},K_{RE}^{I}\right)$, $\left(g_{LK},K_{RE}^{Ca}\right)$, $\left(g_{LK},K_{RE}^{N_{rr}}\right)$.

Next, we check whether our results can be generalized within a certain range of parameters. The above bifurcation analysis allows us to further distinguish different dynamical state regions in the two-parameter space (for example, see Figure 5A). Moreover, the power spectral analysis is applied to estimate the dominant frequency (Df) and second dominant frequency (Sec-Df) of neural oscillations from the time series of *V*_*t*_. Figure 4 illustrates the box-plots of Df and Sec-Df with respect to three oscillations (fast, slow and spindle). It is clear from Figure 4 that the Sec-Df has better differentiation capability than Df. Therefore, the corresponding Sec-Df regions are drawn for each pair of parameters (for example, see Figure 5B) in the following analysis.

Figures 5A,B illustrate the dynamical state regions and Sec-Df regions with 41 × 41 grids in the space of $g_{LK}\times K_{RE}^{I}\in \left[0.018,0.055\right]\times \left[1,9\right]$. As shown in Figure 5A, four different state regions are displayed, whose identification is same as above [i.e., low firing (I), spindle (II), fast oscillation (III) and slow oscillation (IV)]. It can be observed that along with $K_{RE}^{I}$ rising, the model generates more spindle oscillation pattern after lasting a certain fast oscillation period (see the black arrow). By combining the results of frequency analysis shown in Figure 5B, we can outline the spindle oscillation region that falls into the 11–14 Hz frequency range. It should be noted that, the obtained result is consistent with the frequency range [11, 16]Hz characteristic of sleep spindles.

Similar results are obtained in the spaces of $g_{LK}\times K_{RE}^{Ca}$ and $g_{LK}\times K_{RE}^{N_{rr}}$, which are illustrated in Figures 5C–F. We observe that increasing $K_{RE}^{I}$ promotes the generation of spindle oscillations in our model (see the black solid arrow in Figure 5A), while increasing $K_{RE}^{Ca}$ (or $K_{RE}^{N_{rr}}$) suppresses generation of spindle oscillations (see the black dash arrow in Figures 5C,E). These results are in line with the results mentioned above.

**Figure 6:**
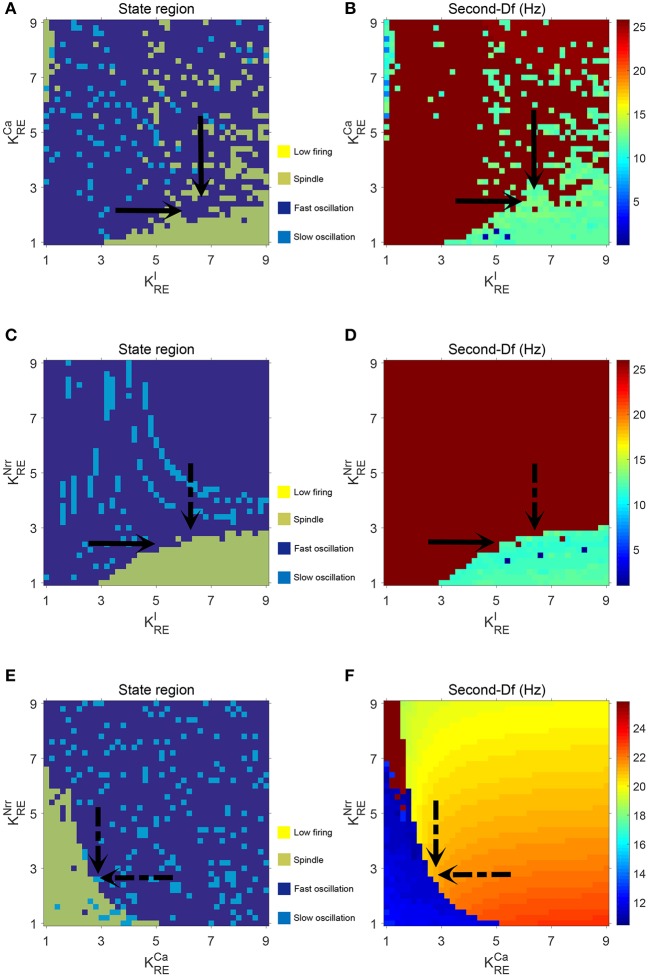
The dynamical state regions **(A,C,E)** and Sec-Df regions **(B,D,F)** in three panels ($K_{RE}^{I}$, $K_{RE}^{Ca}$), ($K_{RE}^{I}$, $K_{RE}^{Nrr}$), and ($K_{RE}^{Ca}$, $K_{RE}^{Nrr})$.

Additionally, in order to demonstrate the interactions among parameters $K_{RE}^{I},K_{RE}^{Ca}$, and $K_{RE}^{Nrr}$, the dynamical state regions and Sec-Df regions for each pairwise combination of them are shown in Figure 6. As expected, both state and Sec-Df analysis in three panels ($K_{RE}^{I}$, $K_{RE}^{Ca}$), ($K_{RE}^{I}$, $K_{RE}^{Nrr}$), and ($K_{RE}^{Ca}$, $K_{RE}^{Nrr}$) provide the same evidence as the above description, that is, the spindle oscillation is generated as $K_{RE}^{I}$ increases (or as $K_{RE}^{Ca}$, $K_{RE}^{Nrr}$ decreases).

### 3.2. The Oscillatory Phenomena Due to Cholinergic Modulation in the Thalamocortical System

Here we concentrate on the effects of cholinergic modulation on thalamocortical rhythms during NREM sleep, including K-complexes, spindles, and slow oscillations. We fix the values of the four parameters to be *g*_*LK*_ = 0.034, $K_{RE}^{I}=5.2$, $K_{RE}^{Ca}=1.5$, and $K_{RE}^{Nrr}=2.1$. Based on the results obtained in the proceeding subsection, we know that spindle oscillations can be generated in the thalamus module with such settings.

**Figure 7:**
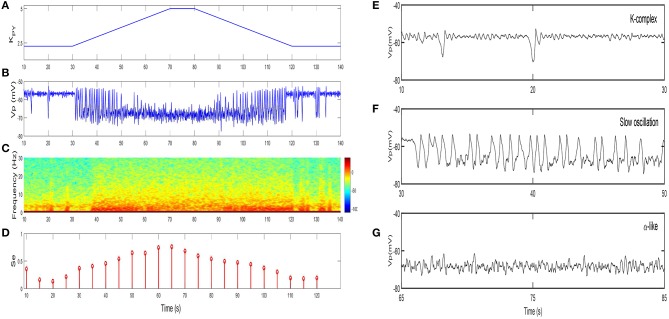
The dynamical behavior of model TC-ACH with the variation of *K*_*PY*_: **(A)** the variation of *K*_*PY*_ over time *t*; **(B)** the model output *V*_*p*_; **(C)** the spectrogram of *V*_*p*_; **(D)** the sample entropy of *V*_*p*_; **(E)** the enlarged model output in [10,30]s (including K-complex); **(F)** the enlarged model output in [30,50]s (including slow oscillation); **(G)** the enlarged model output in [65,85]s (including α-like oscillation).

We first show the dynamical behavior of our TC-ACH model with variation of *K*_*PY*_, which is the strength of the cholinergic projection from CH to PY. In this case, we assume that there is no cholinergic projection from CH to “PY-TC connection” (*K*_*TP*_ = 0), and we apply linear ramps to increase or decrease the parameter value and observe the effects of continuous changes in *K*_*PY*_ on the model. Figure 7B illustrates the model output *V*_*p*_ as *K*_*PY*_ varies in [2.3, 5] linearly (see Figure 7A). In the beginning, when *K*_*PY*_ = 2.3, the model exhibits K-complex oscillations. Then we can find that a small ascent of *K*_*PY*_ drives a transition from K-complexes to slow oscillations. Furthermore, with the gradual increase of *K*_*PY*_, the slow oscillation is kept for a short period until *K*_*PY*_ reaches its higher threshold. At this time, the slow oscillation is replaced by the α-like oscillation. As *K*_*PY*_ ramps down, the model transitions back into its original state gradually (that is, from α-like to slow oscillations, and then from slow oscillations to K-complexes). The enlarged graphs of three different oscillations in 20 s are displayed in Figures 7E–G.

On the basis of the above observations, we conclude that K-complexes and slow oscillations can be triggered by weakening the strength of the cholinergic projection from CH to cortex, while the emergence of α-like activity, characteristic of REM, requires stronger cholinergic input. Here, if we roughly consider that K-complexes and slow oscillations mainly emerge during NREM sleep and α-like rhythms mostly appear during REM sleep, the obtained results in our work are consistent with the conclusion in Lena et al. (2005), that is, the concentration of ACH is lowest during NREM sleep and highest during wake and REM sleep.

The spectral analysis and the sample entropy extracted from the model output *V*_*p*_ also support this conclusion from another point of view. Figure 7C illustrates the spectrogram of *V*_*p*_. During the first and last 40 s where K-complexes and slow oscillations are emerging, the spectral power of *V*_*p*_ mainly falls within the [0, 5]Hz range; while in the middle stage where α-like rhythms begin to appear, it increases to [0, 10]Hz. These results obviously conform with the frequency ranges of certain sleep rhythms. Besides, the sample entropy extracted from *V*_*p*_ also shows a similar tendency. In detail, it is larger in the α-like stage, but smaller in the K-complex and slow wave oscillation stage (see Figure 7D). Sample entropy measures signal complexity, hence this observation is consistent with the conclusion that the complexity of cortical rhythms are decreased as the deeper sleep state, but increased during REM and wake (Bruce et al., 2009; See and Liang, 2011).

**Figure 8:**
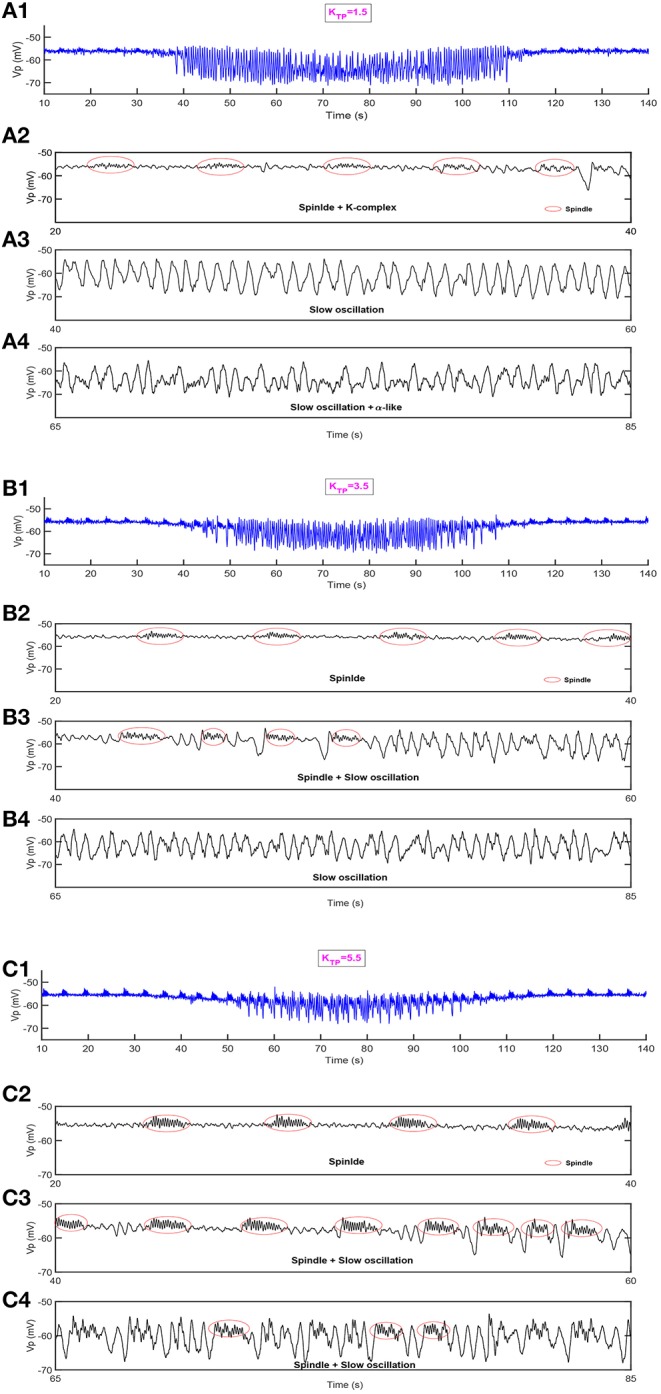
The dynamical behavior of model TC-ACH in the cases where *K*_*TP*_ = 1.5, *K*_*TP*_ = 3.5, *K*_*TP*_ = 5.5. **(A1,B1,C1)** The model output Vp; **(A2,B2,C2)** the enlarged model output in [20,40]s; **(A3,B3,C3)** the enlarged model output in [40,60]s; **(A4,B4,C4)** the enlarged model output in [65,85]s.

Next, we show the dynamical behavior of constructed model TC-ACH with variation of *K*_*TP*_, which is the strength of the cholinergic projection from CH to the connection between PY and TC. Here, *K*_*PY*_ shows the trend as in Figure 7, and *K*_*TP*_ is set to be 1.5, 3.5, 5.5, respectively, in simulations. Figure 8 illustrates the model output *V*_*p*_ under three different cases.

In the first case where the connectivity between TC and PY is regulated by a lower strength of cholinergic projection from CH (i.e., *K*_*TP*_ = 1.5), we observe in Figure 8A1 that some weaker spindle rhythms emerge when the value of *K*_*PY*_ is relatively small. It can be observed more clearly in Figure 8A2 where the enlarged graph exhibits spindle rhythms (with red circle) and K-complex oscillations in [20, 40] s. Meanwhile, in Figures 8A3,A4, we see that the duration of slow oscillation is much longer, and α-like activity is much less by comparing the results shown in Figure 7.

This conclusion can be further verified under other two cases. It can be seen in Figures 8B1–B4 that more spindle rhythms appear as *K*_*PY*_ gradually increases and the α-like activities have been largely replaced by slow waves in the case where *K*_*TP*_ = 3.5. Furthermore, when *K*_*TP*_ is set to be 5.5, the spindle rhythms run through the model output from the beginning to end. We can see from Figure 8C4 that there are still spindle rhythms when *K*_*PY*_ attains its maximum value. These observations reveal that the strength of cholinergic projections from CN to “PY-TC connection” play an important role in promoting the spindle rhythms and prolonging the duration of the NREM state in the thalamocortical system.

## 4. Summary and Discussion

In this paper, we first proposed a novel computational model (TC-ACH) by integrating a neuron population CH into classical thalamo-cortical circuitry (including populations PY, IN, TC, and RE). The connections between five populations are built in accordance with the established mechanisms. Our model considers the neurotransmitter ACH released by neurons in CH, which alters discharge activities of thalamocortical neurons. For simplicity, we represent these additions to the classic model by four cholinergic projections, where coupling coefficients $K_{RE}^{Ca},K_{RE}^{N_{rr}},K_{RE}^{I}$ represent different projections from CH to thalamus, and *K*_*PY*_ and *K*_*TP*_ represent the projections from CH to cortex and thalamocortical system, respectively. On the basis of established model framework of TC-ACH, the corresponding mathematical expression has been formulated in the logic of five populations systematically, where the average membrane potential *V*(*t*) is solved to simulate the rhythms generated by each population.

Next, we applied the developed model TC-ACH to study the effects of ACH modulation on thalamocortical rhythms during NREM sleep in two ways:

1. *Measurement of effects of cholinergic modulation in the thalamus*. In this case, only the thalamic module and projections from CH to RE were considered. Simulation results suggest that cholinergic projection activity is a key factor in modulating oscillation patterns in the thalamic module. Specifically, the model appears to be a stabler spindle oscillation state with the increasing value of $K_{RE}^{I}$, while the growth of $K_{RE}^{Ca}$ (or $K_{RE}^{Nrr}$) leads to reduced spindle rhythms. Moreover, with variation of the potassium leak conductance *g*_*LK*_, which is dramatically modulated by the concentration of ACH (McCormick, 1989, 1992), four different dynamical states including the low firing, spindle, fast oscillation, and slow oscillation can be obtained and transited.
2. *Measurement of effects of cholinergic modulation in the thalamocortical system*. In this case, the dynamical behavior of our TC-ACH model was studied by varying *K*_*PY*_ and *K*_*TP*_, respectively. Simulation results show that the K-complex and slow oscillations can be triggered by weakening strength of *K*_*PY*_, while emergence of α-like activity requires stronger input. Furthermore, when there exists cholinergic input from CH to “PY-TC connection” (that is, *K*_*TP*_ ≠ 0), we found that the duration of oscillation patterns during NREM sleep including K-complexes, spindles and slow oscillations is longer. Additionally, when *K*_*TP*_ is relatively larger, more spindle rhythms appear and α-like activities are largely replaced by slow waves.

It should be noted that a number of electrophysiological experiments have investigated cholinergic modulation of cortex, thalamus or thalamocortical system, respectively (McCormick and Prince, 1986; McCormick, 1989, 1992; Clarke, 2004; Mesulam, 2004; Hasselmo and Giocomo, 2006; Beierlein, 2014). However, few studies correlate ACH modulation with rhythmic activities (Steriade et al., 1993; Steriade, 2004), where the model-based work to study in theory the effects of cholinergic modulation on thalamocortical rhythms during sleep is far less. More than that, what few existing studies are not comprehensive enough in studying the effects of ACH modulation on thalamocortical rhythms, especially during sleep. For example, the effects of ACH modulation are considered only on cortex or thalamus separately, but not on the whole thalamocortical system (Omori and Horiguchi, 2004; Li et al., 2017); the mechanism regarding ACH modulation is only modeled as a certain parameter, but not as a whole neuron population (Li et al., 2017); the model is constructed at the microscopic level, which cannot relate directly to thalamocortical rhythms at the mesoscopic level (Omori and Horiguchi, 2004). Therefore, in order to overcome such limitations, we have constructed a novel computational model (TC-ACH) by incorporating a cholinergic neuron population into the classical thalamo-cortical circuitry at the mesoscopic level. By thus doing, a deeper understanding of the role of cholinergic modulation on thalamocortical system will be got, and further, a critical insight into the mechanisms controlling sleep state may be found.

Besides five projections applied in the modeling (represented by $K_{RE}^{Ca},K_{RE}^{N_{rr}},K_{RE}^{I}$, *K*_*PY*_, and *K*_*TP*_), there still exist cholinergic modulators on other projections, such as corticoreticular and corticothalamic projections. Castro-Alamancos and Calcagnotto (2001) demonstrated that the corticothalamic activity can be filtered by cholinergic activation during arousal high-pass according to the experiments performed *in vitro* and *in vivo*. In addition, as indicated in Itier and Bertrand (2002), the cholinergic modulation on corticoreticular projection may induce the generation of more complex brain rhythms (such as spike and wave) during sleep. However, the quantitative description relating such cholinergic mechanisms to the generation and transition of typical rhythms during NREM sleep is very limited. Therefore, we hope these mechanisms could be further considered with more attempts, to study the cholinergic modulation of thalamocortical rhythms during sleep in different cases.

Another point to stress here is the synaptic connection ways in intra-RE. Previous electrophysiological studies indicated that the thalamic reticular neurons are functionally connected through chemical/electrical synapses (Sanchez-Vives et al., 1997; Landisman et al., 2002; Shu and McCormick, 2002; Long et al., 2004; Deleuze and Huguenard, 2006; Lam et al., 2006). A potentially paradigm-shifting question has been presented as to whether chemical synapses between thalamic reticular neurons are altogether absent in certain mammals or degenerate as a function of increasing age (Landisman et al., 2002; Cruikshank et al., 2010; Hou et al., 2016). However, there still have other works supporting the existence of GABAergic intrareticular synapses. For example, one of the most recent model-based study shows that intrareticular synapses, both chemical and electrical, manifest certain effects on the signal propagation and oscillation (Brown et al., 2019). In our modeling work, by means of considering the chemical synapses in intra-RE population, the measurement of effects of cholinergic modulation in thalamocortical system has been completed well. In contrast, the electrical synapses do not work effectively under the same situation. Consequently, we roughly hypothesize that GABAergic synapses may contribute much more than electrical synapses to the effects of cholinergic modulation in thalamocortical rhythms, at least at the mesoscopic level. But on the other hand, we certainly acknowledge that the electrical intrareticular synapses play important roles vis-à-vis thalamic signaling. Therefore, an important extension of the current work would be the modeling of electrical synapses between reticular neurons from the microscopic point of view, such as the model-based works in Pham and Haas (2018) and Brown et al. (2019).

An important limitation of our TC-ACH model is that it considers only ACH modulation. However, it is known that thalamocortical rhythms during sleep are also directly affected by other neuromodulators, such as noradrenalin (NE), serotonin (5-HT), histamine (HA), and dopamine (DA) from the hypothalamus and brainstem, whose concentrations vary over the night (Lena et al., 2005). Therefore, some apparent questions need to be answered: whether our method can be further developed to shed light on other sleep-related neuromodulators? If so, how to model the corresponding mechanisms and explore their effects on thalamocortical rhythms during sleep? Fortunately, based on the progressive mathematical description of sleep regulatory networks (Kumar et al., 2012; Booth et al., 2017), it may well be possible to carry our model further by constructing a new thalamocortical NMM which contains various sleep-related neuron populations (releasing NE, 5-HT, HA, DA); this is a topic we plan to pursue in future work. In addition, such model-based research can make a contribution to understanding sleep related pathological conditions, such as sleep-related epilepsy.

## Data Availability Statement

The raw data supporting the conclusions of this article will be made available by the authors, without undue reservation, to any qualified researcher.

## Author Contributions

QL, J-LS, S-HL, MW, and RZ designed and performed the research as well as wrote the paper.

### Conflict of Interest

The authors declare that the research was conducted in the absence of any commercial or financial relationships that could be construed as a potential conflict of interest.
